# Murine T Cell‐Dependent Response Affects *Leishmania amazonensis* Amastigote Survival and Virulence

**DOI:** 10.1002/cbin.70197

**Published:** 2026-08-12

**Authors:** Gustavo Henrique Corrêa Soares, Gustavo Rolim Barbosa, Beatriz Simonsen Stolf

**Affiliations:** ^1^ Departamento de Parasitologia, Instituto de Ciências Biomédicas Universidade de São Paulo São Paulo Brasil

**Keywords:** athymic‐nude BALB/c, fitness, *Leishmania amazonensis*, lesion‐derived amastigotes, mouse model, virulence

## Abstract

Our group has previously described the infection course of *Leishmania amazonensis* in BALB/c and BALB/c^nu/nu^ mice, which have distinct T‐cell dependent responses. In this study, lesion‐derived amastigotes exhibited differential protein expression in a host‐dependent manner. Herein, we evaluate the impact of the immune system of these two mouse strains on parasite survival and virulence. For this, BALB/c and BALB/c^nu/nu^ mice were infected with promastigotes of the LV79 strain, and after 13‐weeks of infection, amastigotes were isolated from the lesion footpads. The amastigotes recovered from BALB/c mice were more viable, generating cultures with a higher number of promastigotes than those from BALB/c nude mice. Accordingly, lesion‐derived amastigotes from BALB/c mice displayed higher infectivity, induced larger parasitophorous vacuoles in vitro, and exhibited greater virulence in vivo than those from BALB/c^nu/nu^ mice. These findings demonstrate that the mouse immune environment can shape the biological phenotype of the parasite, directly impacting its survival and virulence.

**Abbreviations**
AMA^BALB/c^
: lesion‐derived amastigotes from BALB/c mice
AMA^nu/nu^
: lesion‐derived amastigotes from BALB/c^nu/nu^ mice
ANOVA: analysis of variance
AUC: area under the curve
BALB/c^nu/nu^
: athymic‐nude BALB/c
BMDM: bone marrow‐derived macrophage
DCL: diffuse cutaneous leishmaniasis
FBS: fetal bovine serum
LCL: localized cutaneous leishmaniasis
LDA: limiting dilution assay
MTT: 3‐[4,5‐dimethyl‐2‐thiazolyl]−2,5‐diphenyl‐2H‐tetrazolium bromide assay
PBS: phosphate‐buffered saline
PS‐like: phosphatidylserine‐like
PV: parasitophorous vacuole
RPMI: Roswell Park Memorial Institute medium
Th1: type 1 helper T cell
Th2: type 2 helper T cell

## 1 Introduction

*Leishmania* are protozoan parasites that cause a group of diseases known as leishmaniasis (World Health Organization 2023). These parasites have a digenetic life cycle, alternating their development between a female phlebotomine sand fly and a vertebrate host (Serafim et al. 2021). Consequently, these parasites face environmental changes that require rapid biological adaptations (Bates and Rogers 2004). Inside the midgut of the sand fly vector, flagellated promastigotes must adapt to lower temperatures, resist digestive proteases and escape elimination during the insect defecation (Cecílio et al. 2022). Once inside the mammalian host, amastigotes need to survive within the host cell and thrive in the acidic, nutrient‐deprived environment of the phagolysosome and the antimicrobial defense of immune system (Carneiro and Peters 2021).

In symptomatic infections, *Leishmania* may cause a wide spectrum of diseases, ranging from skin to visceral lesions (Murray et al. 2005). Different *Leishmania* species can be associated with similar clinical outcomes; furthermore, a single parasite species can cause distinct clinical syndromes (Scorza et al. 2017). This is the case of *L. amazonensis*, in which symptomatic human infections may manifest as a mild disease with localized cutaneous lesions but can also evolve into rare and severe diseases with diffuse and disseminated lesions (Barral et al. 1991; Silveira et al. 2004). This scenario shows that besides the parasite species, the balance of the host immune responses to infection directly contributes to clinical outcome (Scott and Novais 2016).

Mouse models have been extensively employed to investigate cutaneous leishmaniasis and can reproduce some aspects of human infection (reviewed in Loría‐Cervera and Andrade‐Narváez 2014). Host factors that regulate immune response have been identified in mouse infections with *L. major*, in which BALB/c mice mount a predominantly Th2‐biased response and fail to control the infection, whereas C57BL/6 mice develop an effective Th1 response that promotes lesion healing (Scott et al. 1988; Heinzel et al. 1989; Sacks and Noben‐Trauth 2002). However, this dichotomy is not observed in *L. amazonensis* infections, to which most mouse strains are susceptible (Pereira and Alves 2008; de Oliveira Cardoso et al. 2010); Velasquez et al. 2016; Dos‐Santos et al. 2019; Carrara and Stolf 2024). Indeed, infection with *L. amazonensis* induce mixed immune responses, leading to variable degrees of pathology among inbred mouse strains (Pereira and Alves 2008). The impact of T‐cell dependent response on lesion development following *L. amazonensis* infections was previously demonstrated by our group through a comparative analysis of infections in athymic‐nude BALB/c (BALB/c^nu/nu^) and BALB/c mice (Teixeira et al. 2015; Velasquez et al. 2016).

We and others showed that the host immune system not only controls parasite burden but also modulates parasite‐associated factors. We identified parasite proteins that are modulated by the mouse T‐cell response, including proteases and proteins involved in cellular stress responses (Texeira et al. 2015). The exposure of phosphatidylserine‐like (PS‐like) molecules on the surface of amastigote is also modulated by the host immune system. *L. amazonensis* amastigotes derived from BALB/c mice exhibit higher PS‐like exposure compared to those from C57BL/6 mice (Wanderley et al. 2006). Similarly, increased PS‐like exposure was reported in amastigotes from patients with diffuse cutaneous leishmaniasis (DCL) compared with those from localized cutaneous leishmaniasis (LCL) (França‐Costa et al. 2012).

Together, these findings demonstrate that the profile of *L. amazonensis* molecules is influenced by the host background. However, little is known about how the host immune response directly affects other parasite phenotypes. Thus, we investigate the impact of the immune system of two different mouse strains on parasite fitness. By comparing amastigotes from BALB/c and BALB/c^nu/nu^ footpad lesions, we demonstrate that the murine T cell‐dependent response affects *L. amazonensis* amastigote survival and virulence.

## 2 Materials and Methods

### 2.1 Mice

Four‐ to 8‐week‐old female BALB/c and BALB/c^nu/nu^ mice were purchased from the Animal facility of Faculty of Medicine, University of São Paulo (São Paulo, Brazil). Mice were kept at the Animal facility at the Department of Parasitology and Microbiology, Institute of Biomedical Sciences, University of São Paulo (ICB/USP), with food and water *ad libitum*. All experimental procedures were performed according to the guidelines of Brazilian College of Animal Experimentation and the Institutional Animal Care and Use Committee (CEUA) of the ICB/USP under the number #9829290419.

### 2.2 Parasite Culture

*Leishmania amazonensis* LV79 strain (MPRO/BR/1972/M1841), originally isolated from the spiny rat (Proechimys sp.) in Northern Brazil (Chance et al. 1974), was used in this study. Promastigotes were grown at 24°C ± 1°C in complete M199 medium (M199 medium supplemented with HEPES 40 mM [pH 7.4], 0.3 g/L sodium bicarbonate, adenine 100 µM, hemin 5 ppm, 10% heat‐inactivated fetal bovine serum (FBS) and 20 µg/mL gentamicin). Promastigotes were maintained by regular subculturing and used up to the sixth passage for in vivo infection.

### 2.3 Isolation of Lesion‐Derived Amastigotes

BALB/c and BALB/c^nu/nu^ mice, referred to as donor mice, were infected in the left hind footpads with 2 × 10^6^ early stationary‐phase promastigotes (Texeira et al. 2015). After 13 weeks of infection the mice were euthanized, and the paw lesions were removed for amastigote isolation, as previously described (Wanderley et al. 2006). Briefly, lesions were homogenized in sterile PBS using glass tissue grinder tube to ensure complete tissue disruption. The samples were centrifuged at 50*g* for 10 min at 4°C, and the supernatant recovered was centrifuged at 1.450*g* for 17 min at 4°C. The pellet was washed three times with PBS using the same centrifugation conditions. The pellet was resuspended with RPMI medium supplemented with 4% heat‐inactivated FBS and kept under rotation for 3 h at room temperature. The samples were centrifuged, and the pellet obtained was resuspended in 2 mL of ACK lysing buffer (Gibco) and incubated for 2 min on ice. Lesion‐derived amastigotes were washed twice with PBS, and the parasite density was determined using a hemocytometer. Amastigotes were used immediately after isolation in all in vitro and in vivo infection experiments.

### 2.4 Estimate of Cell Viability

Cell viability was estimated by the MTT colorimetric assay as described by De de Rezende et al. (2017). Briefly, 2 × 10^7^ lesion‐derived amastigotes in PBS supplemented with 5 mM glucose were plated in 96‐well plates (Costar) incubated with 5 mg/mL MTT (3‐[4,5‐dimethyl‐2‐thiazolyl]−2,5‐diphenyl‐2H‐tetrazolium bromide) (Sigma‐Aldrich) for 3 h and the reaction was stopped with 10% sodium dodecyl sulfate. The optical density was determined in the FLUOstar Omega Microplate Reader using a test wavelength of 595 nm and reference wavelength of 690 nm. Comparative cell viability (%) was calculated using the following formula: (sample absorbance *100/mean absorbance of the AMA^BALB/c^). Three independent experiments were performed in triplicates.

### 2.5 In Vitro Conversion of Amastigotes‐to‐Promastigotes

Lesion‐derived amastigotes from donor mice were cultivated in complete M199 medium in 24‐well plates (Costar) at 24°C, at densities of 10^3^, 10^4^, and 10^5^ per well. After 4‐days of culture, the density of flagellated promastigotes was determined by counting with a hemocytometer. Three independent experiments were performed in triplicates.

### 2.6 In Vitro Macrophage Infection

Bone marrow‐derived macrophages (BMDM) were obtained from BALB/c mice as previously described (Zamboni and Rabinovitch 2003). BMDM were plated at 4 × 10^5^ cells per well in RPMI medium with 10% heat‐inactivated FBS and 20 µg/mL gentamicin on coverslips in 24‐well plate and incubated overnight in a 5% CO_2_ atmosphere at 37°C. Macrophages were infected with lesion‐derived amastigotes from donor mice at a ratio of 2:1 (parasites:cell) in a 5% CO_2_ atmosphere at 33°C. After 4 h, non‐internalized parasites were removed by PBS‐washing and RPMI medium was added. The plates were incubated under the same conditions for 20 h. Cells were fixed with methanol‐PBS (1:1) and stained with the Instant Prov kit (NewProv). The number of infected‐cells and the total of amastigotes was determined by counting one hundred macrophages per coverslip. The size of parasitophorous vacuoles (PV) was determined as described by França‐Costa et al. (2012) using ImageJ software (version 1.54 g; National Institute of Health, Bethesda, MD, USA). Three independent experiments were performed in triplicate.

### 2.7 In Vivo Cross‐Infection With Lesion‐Derived Amastigotes

BALB/c and BALB/c^nu/nu^ were infected in the left hind footpads with 2×10^6^ lesion‐derived amastigotes recovered from each donor mice. Figure 3a illustrates the workflow of the infection protocol. The thickness of the lesions was measured once a week with a digital caliper (Mitutoyo Corporation, Japan).

### 2.8 Limiting Dilution Assay (LDA)

After 8 weeks of infection, parasite burden was quantified by LDA (Velasquez et al. 2016). Three mice per group were selected and their lesions were collected, pooled and processed as described in Section 2.3. Lesion‐derived parasite suspensions were prepared in 3 mL of complete M199 medium (1 mL per mouse). Following this, 200 µL of the pooled suspension were serially diluted (1:10) in quadruplicate in 96‐well plates (Costar). Plates were examined after 10 days of incubation using an inverted microscope to determine the highest positive dilution, defined as the last well containing viable parasites. Parasite load was then estimated per mouse and multiplied by a factor of 5 to account for the use of only one‐fifth of the total suspension volume.

### 2.9 Statistical Analysis

Data analysis was performed using the GraphPad Prism 8.0.2 software (GraphPad Software Inc, San Diego, CA). The normality of the data set was tested using Shapiro–Wilk test. The unpaired *t*‐test with Welch's correction was used to compare data from lesion‐derived amastigotes from donor mice. The One‐way analysis of variance (ANOVA) test followed by Tukey's multiple comparison post hoc test was used to compare in vivo infection data. A *p‐value* < 0.05 was considered significant for all analysis.

## 3 Results and Discussion

**Figure 1:**
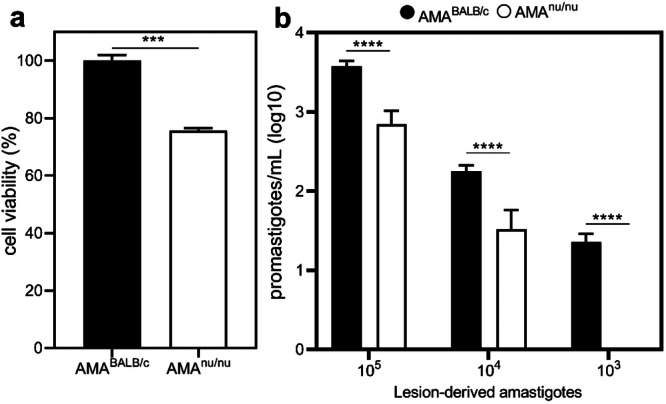
Survival of lesion‐derived amastigotes from BALB/c and BALB/c^nu/nu^ donor mice. (a) Comparative cell viability (%) estimated by MTT assay and (b) In vitro conversion of lesion‐derived amastigotes into promastigotes (data presented as log10). Values are expressed as mean ± standard deviation. Statistical analysis was performed using an unpaired *t*‐test. ***p < 0.001 and ****p < 0.0001. Data shown are from one representative experiment out of three independent experiments with similar profiles.

It was previously reported by our group that footpad infections by the *L. amazonensis* LV79 strain have different characteristics in BALB/c and BALB/c^nu/nu^ mice (Velasquez et al. 2016). Proteome analysis of footpad lesion‐derived amastigotes from these mice revealed that ~3% of the proteins were differentially expressed, indicating that the amastigote's protein expression is modulated in a host‐dependent manner (Texeira et al. 2015). This data prompted us to investigate whether the immune system of these mice would also affect other characteristics of the parasite, such as their survival and virulence. To test this, we infected BALB/c and BALB/c^nu/nu^ mice with LV79 promastigotes and followed the infection for 13 weeks (data not shown). After this period, we recovered the lesion‐derived amastigotes from each donor mice, and their metabolic activity, used here as an estimate of viability, was determined using the MTT assay. The amastigotes recovered from BALB/c were more viable compared to those from BALB/c^nu/nu^ mice (*p* < 0.001) (Figure 1a). Lesion‐derived amastigotes were monitored for conversion to flagellated promastigotes and, accordingly, lesion‐derived amastigotes from BALB/c mice were more efficient in the conversion, resulting in cultures with a higher number of promastigotes (Figure 1b).

**Figure 2:**
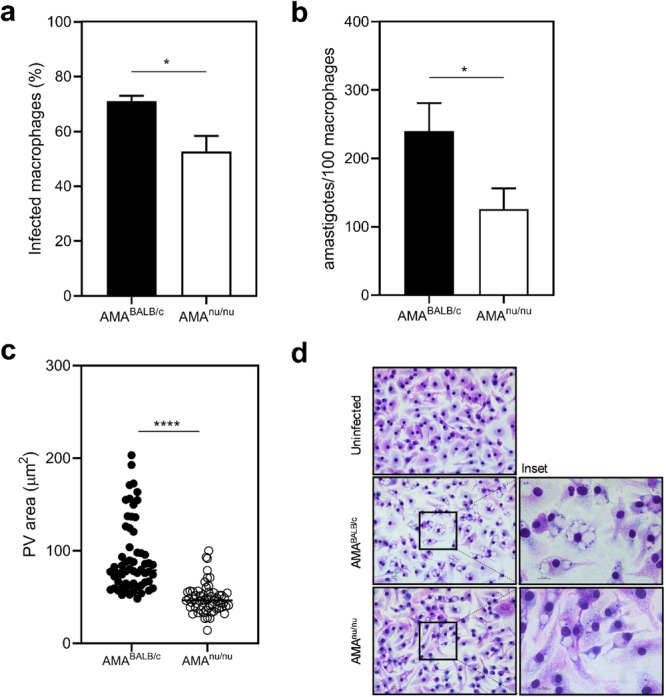
In vitro infectivity of lesion‐derived amastigotes from BALB/c and BALB/c^nu/nu^ donor mice. (a) Percentage of infected macrophages; (b) Counting of intracellular amastigotes/100 macrophages; (c) Parasitophorous vacuole (PV) analysis; and (d) Photomicrograph of infected macrophages. Scale‐bar: 10 µm. Values are expressed as mean ± standard deviation. Statistical analysis was performed using an unpaired *t*‐test. *p < 0.05 and ***p < 0.001. Data shown are from one representative experiment out of three independent experiments with similar profiles.

We next evaluated the in vitro infectivity of recovered amastigotes from donor mice in BMDM. Amastigotes from BALB/c mice were more infective compared with those from BALB/c^nu/nu^ mice (Figure 2). The percentage of infected macrophages was significantly higher at 24 h with amastigotes from BALB/c (71 ± 2%) compared to those from BALB/c^nu/nu^ (52.6 ± 5.6%) (*p* < 0.05) (Figure 2a). Parasite burden determined by intracellular amastigotes counts was also higher for parasites derived from BALB/c mice, confirming the higher infectivity of these amastigotes (*p* < 0.05) (Figure 2b). It has previously been demonstrated that the ability of *L. amazonensis* to induce large PVs is closely associated with increased infectivity (França‐Costa et al. 2012). Based on this, we investigated whether the differential infectivity observed between the recovered amastigotes from the two donor mice would be accompanied by differences in PV size. As shown in Figure 2c, PV size induced by lesion‐derived amastigotes from BALB/c (93.12 ± 39.08 µm^2^) was significantly higher than those from BALB/c^nu/nu^ (48.34 ± 15.81 µm^2^) (*p* < 0.001).

**Figure 3:**
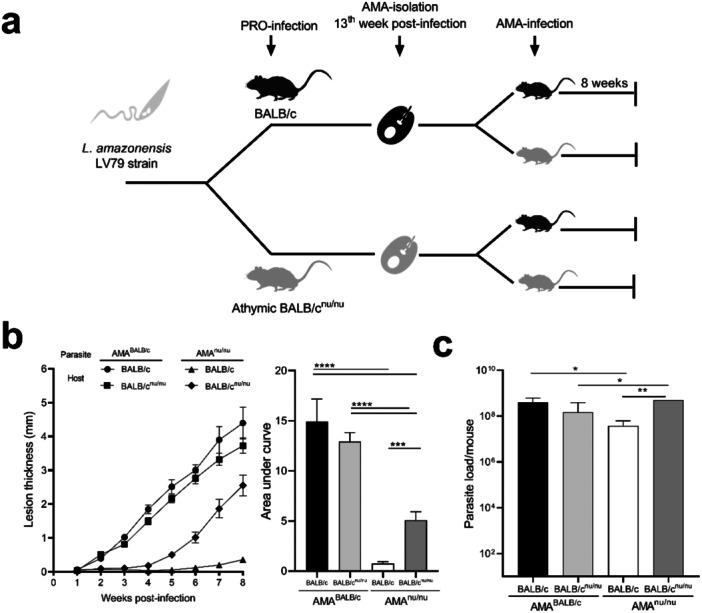
In vivo virulence of lesion‐derived amastigotes from BALB/c and BALB/c^nu/nu^ donor mice. (a) Workflow of the cross‐infection protocol, in which BALB/c and BALB/c^nu/nu^ mice were infected with *Leishmania amazonensis* promastigotes, and thirteen weeks post‐infection, lesion‐derived amastigotes were used to infect new set of BALB/c and BALB/c^nu/nu^ mice; (b) Lesion thickness and area under curve, and (c) Estimated parasite load per mouse at 8 weeks post‐infection. Values are expressed as mean ± standard deviation. Statistical analysis was performed by One‐way ANOVA followed by Tukey's multiple comparison post hoc test. *p < 0.05, **p < 0.01, ***p < 0.001 and ****p < 0.0001. Results from one experiment with five animals per group.

We also assessed the virulence of these parasites using a cross‐infection protocol, in which a new set of mice were infected with lesion‐derived amastigotes from each donor mouse (Figure 3a). Footpad thickness in both BALB/c and BALB/c^nu/nu^ increased earlier when infected with lesion‐derived amastigotes from BALB/c donor mice. From 5 weeks onwards, lesions became apparent in BALB/c^nu/nu^ mice infected with lesion‐derived amastigotes from BALB/c^nu/nu^ donor mice (Figure 3a). The course of infection of each experimental group was compared by area under the curve (AUC). No differences were observed between BALB/c and BALB/c^nu/nu^ infected with amastigotes from BALB/c donors. However, lesions in BALB/c^nu/nu^ infected with amastigotes from BALB/c^nu/nu^ donors were larger compared to those of BALB/c infected with the same parasites (Figure 3b). Parasite burden in lesions was determined 8 weeks post‐infection, a time point at which differences in lesion size among the experimental groups were already evident, allowing a reliable assessment of parasite burden while ensuring animal welfare. As shown in Figure 3c, parasite burden was similar between BALB/c and BALB/c^nu/nu^ mice infected with lesion‐derived amastigotes from BALB/c donors. On the other hand, significant differences were observed in the parasite burden of lesions caused by parasites from BALB/c^nu/nu^ donors. Interestingly, BALB/c^nu/nu^ mice developed higher parasite burdens when infected with parasites from BALB/c^nu/nu^ donor mice compared to BALB/c (Figure 3b), reinforcing our previous statement (Velasquez et al. 2016) that lesion thickness is not a good estimate of parasite load in immunodeficient mice.

The present study used an experimental approach that allowed us to assess the impact of host immune system on parasite fitness at the amastigote stage. Curiously, lesion‐derived amastigotes from BALB/c mice exhibited higher viability, although BALB/c footpad lesions displayed a higher inflammatory cell infiltration compared to those of BALB/c^nu/nu^ mice (Velasquez et al. 2016). This suggests that the stronger immune pressure in BALB/c footpad lesions may select for more viable and infective amastigotes, potentially explaining the phenotypic differences observed.

Our results indicate that amastigotes recovered from BALB/c footpad lesions are also more infective for macrophages in vitro. Moreover, these amastigotes lead to thicker lesions when compared to BALB/c^nu/nu^‐derived parasites. Altogether, these findings demonstrate that the host immune environment, more specifically the T cell‐dependent response, is able to shape and modulate the phenotype of the parasite, directly impacting its survival and virulence.

## Author Contributions

**Gustavo Henrique Corrêa Soares:** investigation and formal analysis, writing – original draft and writing – review and editing. **Gustavo Rolim Barbosa:** investigation and formal analysis and writing – review and editing. **Beatriz Simonsen Stolf:** conceptualization, supervision, validation, writing – original draft, writing – review and editing and funding acquisition.

## Conflicts of Interest

The authors declare no conflicts of interest.

## Data Availability

The data of this study are available from the corresponding author upon reasonable request.

## References

[cbin70197-bib-0001] Barral, A. , D. Pedral‐Sampaio , J. G. Grimaldi , et al. 1991. “Leishmaniasis in Bahia, Brazil: Evidence That *Leishmania amazonensis* Produces a Wide Spectrum of Clinical Disease.” American Journal of Tropical Medicine and Hygiene 44, no. 5: 536–546. 10.4269/ajtmh.1991.44.536. 2063957

[cbin70197-bib-0002] Bates, P. A. , and M. E. Rogers . 2004. “New Insights Into the Developmental Biology and Transmission Mechanisms of *Leishmania* .” Current Molecular Medicine 4, no. 6: 601–609. 10.2174/1566524043360285. 15357211

[cbin70197-bib-0003] Carneiro, M. B. , and N. C. Peters . 2021. “The Paradox of a Phagosome Lifestyle: How Innate Host Cell‐*Leishmania amazonensis* Interactions Lead to a Progressive Chronic Disease.” Frontiers in Immunology 12: 728848. 10.3389/fimmu.2021.728848. 34557194 PMC8452962

[cbin70197-bib-0004] Carrara, G. M. , and B. S. Stolf . 2024. “FVB/NJ Strain as a Mouse Model for Cutaneous Leishmaniasis by *Leishmania (L.) amazonensis* .” Memórias do Instituto Oswaldo Cruz 119: e230182. 10.1590/0074-02760230182. 38511814 PMC10941652

[cbin70197-bib-0005] Cecílio, P. , A. Cordeiro‐da‐Silva , and F. Oliveira . 2022. “Sand Flies: Basic Information on the Vectors of Leishmaniasis and Their Interactions With *Leishmania* Parasites.” Communications Biology 5, no. 1: 305. 10.1038/s42003-022-03240-z. 35379881 PMC8979968

[cbin70197-bib-0006] Chance, M. L. , W. Peters , and L. Shchory . 1974. “Biochemical Taxonomy of *Leishmania*: I: Observations on DNA.” Annals of Tropical Medicine & Parasitology 68, no. 3: 307–316. PMID: 4447389. 4447389

[cbin70197-bib-0007] Dos‐Santos, J. S. , L. Firmino‐Cruz , and T. D. Ramos , et al. 2019. “Characterization of Sv129 Mice as a Susceptible Model to *Leishmania amazonensis* .” Frontiers in Medicine 6: 100. 10.3389/fmed.2019.00100. 31192210 PMC6548835

[cbin70197-bib-0008] França‐Costa, J. , J. L. Wanderley , P. Deolindo , et al. 2012. “Exposure of Phosphatidylserine on *Leishmania amazonensis* Isolates Is Associated With Diffuse Cutaneous Leishmaniasis and Parasite Infectivity.” PLoS One 7, no. 5: e36595. 10.1371/journal.pone.0036595. 22574191 PMC3344919

[cbin70197-bib-0009] Heinzel, F. P. , M. D. Sadick , B. J. Holaday , R. L. Coffman , and R. M. Locksley . 1989. “Reciprocal Expression of Interferon Gamma or Interleukin 4 During the Resolution or Progression of Murine Leishmaniasis. Evidence for Expansion of Distinct Helper T Cell Subsets.” Journal of Experimental Medicine 169, no. 1: 59–72. 10.1084/jem.169.1.59. 2521244 PMC2189187

[cbin70197-bib-0010] Loría‐Cervera, E. N. , and F. J. Andrade‐Narváez . 2014. “Animal Models for the Study of Leishmaniasis Immunology.” Revista do Instituto de Medicina Tropical de São Paulo 56, no. 1: 1. 10.1590/S0036-46652014000100001. 24553602 PMC4085833

[cbin70197-bib-0011] Murray, H. W. , J. D. Berman , C. R. Davies , and N. G. Saravia . 2005. “Advances in Leishmaniasis.” Lancet 366, no. 9496: 1561–1577. 10.1016/S0140-6736(05)67629-5. 16257344

[cbin70197-bib-0012] de Oliveira Cardoso, F. , S. F. da , C. de Souza , et al. 2010. “Immunopathological Studies of *Leishmania amazonensis* Infection in Resistant and in Susceptible Mice.” Journal of Infectious Diseases 201, no. 12: 1933–1940. 10.1086/652870. 20462353

[cbin70197-bib-0013] Pereira, B. A. , and C. R. Alves . 2008. “Immunological Characteristics of Experimental Murine Infection With *Leishmania (Leishmania) amazonensis* .” Veterinary Parasitology 158, no. 4: 239–255. 10.1016/j.vetpar.2008.09.015. 18922635

[cbin70197-bib-0014] de Rezende, E. , R. Kawahara , M. S. Pena , G. Palmisano , and B. S. Stolf . 2017. “Quantitative Proteomic Analysis of Amastigotes From *Leishmania (L.) amazonensis* LV79 and PH8 Strains Reveals Molecular Traits Associated With the Virulence Phenotype.” PLoS Neglected Tropical Diseases 11, no. 11: e0006090. 10.1371/journal.pntd.0006090. 29176891 PMC5720813

[cbin70197-bib-0015] Sacks, D. , and N. Noben‐Trauth . 2002. “The Immunology of Susceptibility and Resistance to *Leishmania major* in Mice.” Nature Reviews Immunology 2, no. 11: 845–858. 10.1038/nri933. 12415308

[cbin70197-bib-0016] Scorza, B. M. , E. M. Carvalho , and M. E. Wilson . 2017. “Cutaneous Manifestations of Human and Murine Leishmaniasis.” International Journal of Molecular Sciences 18, no. 6: 1296. 10.3390/ijms18061296. 28629171 PMC5486117

[cbin70197-bib-0017] Scott, P. , P. Natovitz , R. L. Coffman , E. Pearce , and A. Sher . 1988. “Immunoregulation of Cutaneous Leishmaniasis. T Cell Lines That Transfer Protective Immunity or Exacerbation Belong to Different T Helper Subsets and Respond to Distinct Parasite Antigens.” Journal of Experimental Medicine 168, no. 5: 1675–1684. 10.1084/jem.168.5.1675. 2903212 PMC2189120

[cbin70197-bib-0018] Scott, P. , and F. O. Novais . 2016. “Cutaneous Leishmaniasis: Immune Responses in Protection and Pathogenesis.” Nature Reviews Immunology 16, no. 9: 581–592. 10.1038/nri.2016.72. 27424773

[cbin70197-bib-0019] Serafim, T. D. , I. V. Coutinho‐Abreu , R. Dey , et al. 2021. “Leishmaniasis: The Act of Transmission.” Trends in Parasitology 37, no. 11: 976–987. 10.1016/j.pt.2021.07.003. 34389215

[cbin70197-bib-0020] Silveira, F. T. , R. Lainson , and C. E. Corbett . 2004. “Clinical and Immunopathological Spectrum of American Cutaneous Leishmaniasis With Special Reference to the Disease in Amazonian Brazil: A Review.” Memórias do Instituto Oswaldo Cruz 99: 239–251. 10.1590/s0074-02762004000300001. 15273794

[cbin70197-bib-0025] Teixeira, P. C. , L. G. Velasquez , A. P. Lepique , et al. 2015. “Regulation of *Leishmania* (*L.*) *amazonensis* Protein Expression by Host T Cell Dependent Responses: Differential Expression of Oligopeptidase B, Tryparedoxin Peroxidase and HSP70 Isoforms in Amastigotes Isolated From BALB/c and BALB/c Nude Mice.” PLoS Neglected Tropical Diseases 9, no. 2: e0003411. 10.1371/journal.pntd.0003411. 25692783 PMC4333223

[cbin70197-bib-0021] Velasquez, L. G. , M. K. Galuppo , E. De Rezende , et al. 2016. “Distinct Courses of Infection With *Leishmania (L.) amazonensis* Are Observed in BALB/c, BALB/c Nude and C57BL/6 Mice.” Parasitology 143, no. 6: 692–703. 10.1017/S003118201600024X. 26892342

[cbin70197-bib-0022] Wanderley, J. L. , M. E. Moreira , A. Benjamin , A. C. Bonomo , and M. A. Barcinski . 2006. “Mimicry of Apoptotic Cells by Exposing Phosphatidylserine Participates in the Establishment of Amastigotes of *Leishmania (L.) amazonensis* in Mammalian Hosts.” Journal of Immunology 176, no. 3: 1834–1839. 10.4049/jimmunol.176.3.1834. 16424214

[cbin70197-bib-0023] World Health Organization . “Leishmaniasis [Internet].” 2023. https://www.who.int/news-room/fact-sheets/detail/leishmaniasis.

[cbin70197-bib-0024] Zamboni, D. S. , and M. Rabinovitch . 2003. “Nitric Oxide Partially Controls *Coxiella burnetii* Phase II Infection in Mouse Primary Macrophages.” Infection and Immunity 71, no. 3: 1225–1233. 10.1128/iai.71.3.1225-1233.2003. 12595436 PMC148841

